# Andrographolide Modulates Fibrogenic and Oxidative Stress Responses in Human Lung Fibroblasts

**DOI:** 10.1002/kjm2.70218

**Published:** 2026-04-22

**Authors:** Yu‐Hsin Tseng, Yen‐Hsien Wu, Yi‐Ching Liu, I‐Chen Chen, Chung‐Yu Yeh, Shang‐En Huang, Ren‐Long Jan, Jwu‐Lai Yeh, Jong‐Hau Hsu

**Affiliations:** ^1^ Department of Pediatrics Kaohsiung Medical University Hospital, Kaohsiung Medical University Kaohsiung Taiwan; ^2^ Department of Pediatrics, School of Medicine, College of Medicine Kaohsiung Medical University Kaohsiung Taiwan; ^3^ Department of Pharmacology, College of Medicine Kaohsiung Medical University Kaohsiung Taiwan; ^4^ Department of Seafood Science National Kaohsiung University of Science and Technology Kaohsiung Taiwan; ^5^ Department of Pediatrics Chi Mei Medical Center Tainan Taiwan; ^6^ Graduate Institute of Medicine Kaohsiung Medical University Kaohsiung Taiwan; ^7^ Department of Medical Research Kaohsiung Medical University Hospital Kaohsiung Taiwan; ^8^ Department of Marine Biotechnology and Resources National Sun Yat‐Sen University Kaohsiung Taiwan

**Keywords:** andrographolide, fibroblasts, glutathione, pulmonary fibrosis, transforming growth factor‐β1

## Abstract

Pulmonary fibrosis is a progressive lung disorder characterized by fibroblast activation and excessive extracellular matrix deposition. Andrographolide (ANDRO) has been reported to attenuate pulmonary fibrosis, but the underlying molecular mechanisms remain incompletely understood. In this study, we investigated the effects of ANDRO on transforming growth factor‐beta 1 (TGF‐β1)‐induced fibrogenesis in human lung fibroblasts. Comprehensive transcriptomic analysis revealed that ANDRO reversed TGF‐β1‐perturbed genes associated with fibrogenesis and glutathione (GSH) metabolism. Functionally, ANDRO restored TGF‐β1‐inhibited total GSH levels and suppressed TGF‐β1‐induced reactive oxygen species (ROS) accumulation. Together, these findings identify GSH metabolism as a previously unrecognized mechanism underlying the antifibrotic effects of ANDRO in human lung fibroblasts.

## 1 Introduction

Pulmonary fibrosis is the terminal stage of various interstitial lung diseases or pulmonary infections [1, 2, 3]. Despite substantial research efforts, only two drugs—pirfenidone and nintedanib—have been approved by the US Food and Drug Administration (FDA) for clinical use, both of which primarily delay the decline in lung function rather than reverse fibrosis [4, 5]; however, these drugs have limitations, including high cost, side effects, and incompletely understood mechanisms [6, 7], so exploring the molecular mechanisms of potential antifibrotic agents such as ANDRO is critical for identifying new therapeutic strategies for pulmonary fibrosis.

Pathological fibrogenesis is a dynamic and multifactorial process involving crosstalk among fibroblasts, epithelial cells, immune cells and endothelial compartments [8]. Fibroblasts, the predominant mesenchymal cell type in connective tissues, are essential for tissue remodeling and wound repair [9]. Upon injury, fibroblasts become activated, proliferate, and differentiate into myofibroblasts—a process marked by α‐smooth muscle actin expression and enhanced contractility [10]. Persistent activation of myofibroblasts leads to excessive extracellular matrix (ECM) production, scar formation, and structural remodeling of lung tissue [11, 12]. In idiopathic pulmonary fibrosis (IPF), the most common form of idiopathic interstitial pneumonia, the persistent presence of myofibroblasts within fibroblastic foci results in abnormal deposition of collagen, elastin, and fibronectin, ultimately causing alveolar collapse and progressive loss of pulmonary function [3, 13].

Transforming growth factor‐β1 (TGF‐β1) is recognized as a master profibrogenic cytokine that drives myofibroblast differentiation and ECM synthesis in nearly all fibrotic diseases [14, 15, 16]. However, direct inhibition of TGF‐β1 signaling is not a viable therapeutic strategy due to its pleiotropic physiological roles and potential adverse effects [17]. Thus, identifying downstream modulators of TGF‐β1 signaling has emerged as a promising approach for developing targeted antifibrotic therapies.

Andrographolide (ANDRO), a diterpenoid derived from *Andrographis paniculata* , exhibits multiple pharmacological activities, including anti‐inflammatory, antioxidant, and antiepithelial–mesenchymal transition (anti‐EMT) effects [18]. ANDRO has been shown to activate endogenous antioxidant signaling pathways such as Nrf2/HO‐1 via p38 MAPK and ERK, which enhances cellular defenses against oxidative stress [19]. In addition, ANDRO mitigates oxidative damages by modulating the PI3K/AKT/mTOR and NRF2 pathways, leading to reduced reactive oxygen species (ROS) generation in vitro and in vivo [20, 21]. Furthermore, previous studies have demonstrated that ANDRO protects against pulmonary fibrosis in rodent models by inhibiting inflammation, oxidative stress, EMT, and fibroblast activation [22, 23, 24]. However, the precise molecular mechanisms in human lung fibroblasts remain unclear, particularly regarding the pathways linking ANDRO to redox regulation and fibrogenesis.

In this study, we employed RNA‐seq‐based transcriptomic profiling in TGF‐β1‐stimulated human lung fibroblasts, which revealed glutathione (GSH) metabolism as a key pathway modulated by ANDRO. Subsequent molecular analyses confirmed the role of GSH metabolism in its antifibrotic effects. These findings reveal the molecular mechanisms underlying ANDRO's antifibrotic effects, providing a rationale for the development of natural product‐derived therapies.

## 2 Methods

### 2.1 Cell Culture and Drug Treatment

MRC5 (Cat. No. CCL‐171) and WI‐38 (Cat. No. CCL‐75) cells were purchased from the American Type Culture Collection (ATCC, Manassas, VA, USA) and were cultured in Eagle's Minimum Essential Medium (Cat. No. 30‐2003, *ATCC*) supplemented with 10% fetal bovine serum (Gibco, Amarillo, TX, USA) and 1% antibiotic–antimycotic agents (Gibco). Cells were cultured in a humidified incubator at 37°C with 5% CO_2_ and subcultured at approximately 80% confluence. TGF‐β1 (Cat. No. T7039) and ANDRO (Cat. No. AL‐365645) were purchased from Sigma Aldrich (St. Louis, MO, USA).

### 2.2 High‐Throughput RNA Sequencing (RNA‐Seq)

Total RNA was extracted with TRIzol reagent (Thermo Fisher Scientific, Waltham, MA, USA) and submitted to Genomics, BioSci & Tech Co. (New Taipei City, Taiwan) for sequencing. Libraries was prepared using the TruSeq Stranded mRNA Library Prep Kit (Illumina, San Diego, CA, USA). Briefly, mRNA was purified from 1 μg of total RNA with oligo(dT)‐coupled magnetic beads and fragmented into short fragments. First‐strand cDNA was synthesized using reverse transcriptase and random primers. Adaptors were ligated and purified using the AMPure XP system (Beckman Coulter, Beverly, USA). Double‐strand cDNA was then generated and adenylated at the 3′ ends. Qualified libraries were sequenced with 150‐bp paired‐end reads on the Illumina NovaSeq 6000 platform.

### 2.3 Bioinformatics Analysis

Low‐quality bases and adapter sequences in raw data were removed using the fastp program (version 0.20.0). The filtered reads were aligned to reference genomes using HISAT2 (version 2.1.0). Gene abundance was quantified using FeatureCounts (version 2.0.1). Differentially expressed genes (DEGs) were identified using EdgeR (version 3.36.0), and functional enrichment analysis was performed using ShinyGO (version 0.76), and protein–protein interaction networks among the candidate genes was predicted using STRING (version 11.5).

### 2.4 Reverse Transcription Quantitative PCR (RT‐qPCR)

Total RNA was extracted using the TRIzol total RNA extraction kit (Thermo Fisher Scientific Inc.), and cDNA was synthesized using the qScriberTM cDNA synthesis kit (highQu GmbH, Kraichtal, Germany). The reverse transcription process involved incubation at 42°C–50°C for 30 min to synthesize cDNA, followed by enzyme inactivation at 85°C for 10 min. PCR amplification reactions were set up in 10‐μL volumes with primers and Fast SYBR Green Master mix (Thermo Fisher Scientific Inc.) and performed on the ABI 7500 system (Applied Biosystems; Thermo Fisher Scientific Inc.). The thermal cycling conditions of PCR were as follows: 95°C for 20 s, 40 cycles of 95°C for 3 s, and 60°C for 30 s. GAPDH was used as the internal control. Primer sequences used for RT‐qPCR are listed in Table 1.

**TABLE 1:** Sequences of primers used for RT‐qPCR.

Gene symbol	Forward primer (5′→3′)	Reverse primer (5′→3′)
*ACTA2*	CTATGCCTCTGGACGCACAACT	CAGATCCAGACGCATGATGGCA
*FN1*	ACAACACCGAGGTGACTGAGAC	GGACACAACGATGCTTCCTGAG
*CDH2*	CATCCAGACCGACCCAAACA	ACAGACACGGTTGCAGTTGA
*GCLM*	CTGCGGTATTCGGTCATTGTG	TGACACCATTTACAGGCAGT
*GSR*	CCGAAAACTTGCCCATCGAC	GCTGAAGACCACAGTTGGGA
*G6PD*	CTACCGCATCGACCACTACC	TGTTGTCCCGGTTCCAGATG
*GAPDH*	CTGGGCTACACTGAGCACC	AAGTGGTCGTTGAGG GCAATG

### 2.5 Western Blotting

Cells were lysed in RIPA buffer supplemented with protease inhibitor and phosphatase inhibitor (Thermo Fisher Scientific), then after centrifugation at 13,000 *g* for 15 min at 4°C, the supernatant containing total proteins was collected and protein concentrations were quantified using the Pierce BCA protein assay kit (Thermo Fisher Scientific). Equal amounts of protein were separated on 4%–12% Bolt Bis‐Tris Plus gels (Invitrogen, Carlsbad, CA, USA) and transferred to PVDF membranes. The membranes were blocked in 5% bovine serum albumin containing 0.1% Tween‐20 for 1 h at room temperature, followed by overnight incubation at 4°C with primary antibodies against α‐SMA (Cat. No. NBP2‐33006, Novus Biologicals, Centennial, CO, USA), Fibronectin (Cat. No. NBP1‐91258, Novus Biologicals), N‐cadherin (Cat. No. 13116, Cell Signaling Technology, Danvers, MA, USA), GCLM (Cat. No. GB111827, Servicebio, Wuhan, China), G6PD (Cat. No. E‐AB‐66188, Elabscience, Wuhan, China), GSR (Cat. No. E‐AB‐10354, Elabscience), and GAPDH (Cat. No. NB300‐221, Novus Biologicals). After washing, the membranes were incubated with horseradish peroxidase–conjugated secondary antibodies (1:10,000, GE Healthcare, Madison, WI, USA) for 1 h at room temperature. Protein bands were visualized using an enhanced chemiluminescence detection kit (Cat. No. RPN2235, Cytiva, Shanghai, China) according to the manufacturer's instructions.

### 2.6 GSH Assay

GSH levels were detected using the GSH assay kit (Cat. No. MAK440, Sigma Aldrich) in accordance with the manufacturer's instructions. Cells (2 × 10^6^) were washed with 1× PBS and centrifuged at 900 *g* for 5 min at room temperature. After the removal of PBS, the cells were lysed by sonication in 200 μL of cold buffer containing 50 mM phosphate, pH 7, and 1 mM EDTA, then the cell lysates were centrifuged at 10,000 *g* for 15 min at 4°C. Next, the supernatant was transferred to a clean tube, the deproteination step was performed using meta‐phosphoric acid solution, then 100 μL of the working reagent was added to each sample and standard well. The optical density of each well was read at 412 nm at 0 (OD0Min) and 10 min (OD10Min) after mixing, with the ΔOD calculated by subtracting the OD0Min reading from the OD10Min reading for each standard and sample and the slope of the standard curve determined by using linear regression. Total GSH levels in the samples were then calculated using the following formula: [(ΔOD_Sample_ − ΔOD_Blank_)/slope (μM^−1^)] × 150.

### 2.7 2′,7′‐Dichlorodihydrofluorescein Diacetate Staining

Intracellular ROS levels were detected using 2′,7′‐dichlorodihydrofluorescein diacetate (Cat. No. 10058, DCFH‐DA, Biotium Inc., Fremont, CA, USA), an oxidative‐sensitive fluorescent probe. Cells were stained with 40 μM DCFH‐DA for 1 h at 37°C in the dark. Fluorescence intensity was immediately measured using a microplate reader at an excitation/emission wavelength of 488/529 nm. Results were expressed as relative fluorescence intensity normalized to the control. Subsequently, cell viability was assessed using the Cell Counting Kit‐8 (Cat. No. CC02‐20, Energenesis‐biomedical co. Ltd. [Topcells], Taipei, Taiwan), and ROS production data were normalized to cell viability.

### 2.8 Statistical Analysis

Data were analyzed using GraphPad Prism5.0 software (GraphPad Software Inc., San Diego, CA, USA), with data represented as the mean ± standard error (SEM) of three independent experiments. Due to the small sample size (*n* = 3), formal tests for normality were not performed. One‐way ANOVA was used to *compare* differences among groups, followed by Tukey's multiple comparisons test for post hoc analysis, whereas Student's *t*‐test was used to *compare* differences between two groups as indicated in the figure legends. A *p* value below 0.05 was considered statistically significant.

## 3 Results

### 3.1 ANDRO Reversed TGF‐β1‐Modulated the Expression of Genes and Proteins Associated With Lung Fibrogenesis

To confirm the antifibrotic effect of ANDRO, genes associated with fibrogenesis such as *ACTA2* (encoding alpha‐smooth muscle actin, α‐SMA), *FN1* (encoding fibronectin), and *CDH2* (encoding N‐cadherin) were examined together with their corresponding proteins. MRC5 cells were pretreated with or without ANDRO (10 μM) for 4 h, followed by treatment with exogenous TGF‐β1 (4 ng/mL) for 48 h. Gene and protein expression levels were measured by RT‐qPCR and Western blotting, respectively. The results demonstrated that ANDRO significantly attenuated TGF‐β1‐induced upregulation of *ACTA2*, *FN1*, and *CDH2*, as well as the levels of their corresponding proteins (Figure 1). It is worth noting, in the absence of TGF‐β1 stimulation, RT‐qPCR analysis showed that ANDRO significantly suppressed the basal expression of *ACTA2, FN1*, *and CDH2* in MRC5 cells. In parallel experiments using WI‐38 cells, ANDRO significantly inhibited the expression of *ACTA2* and *FN1*, while *CDH2* showed a slight downward trend that did not reach statistical significance (Figure S1).

**FIGURE 1:**
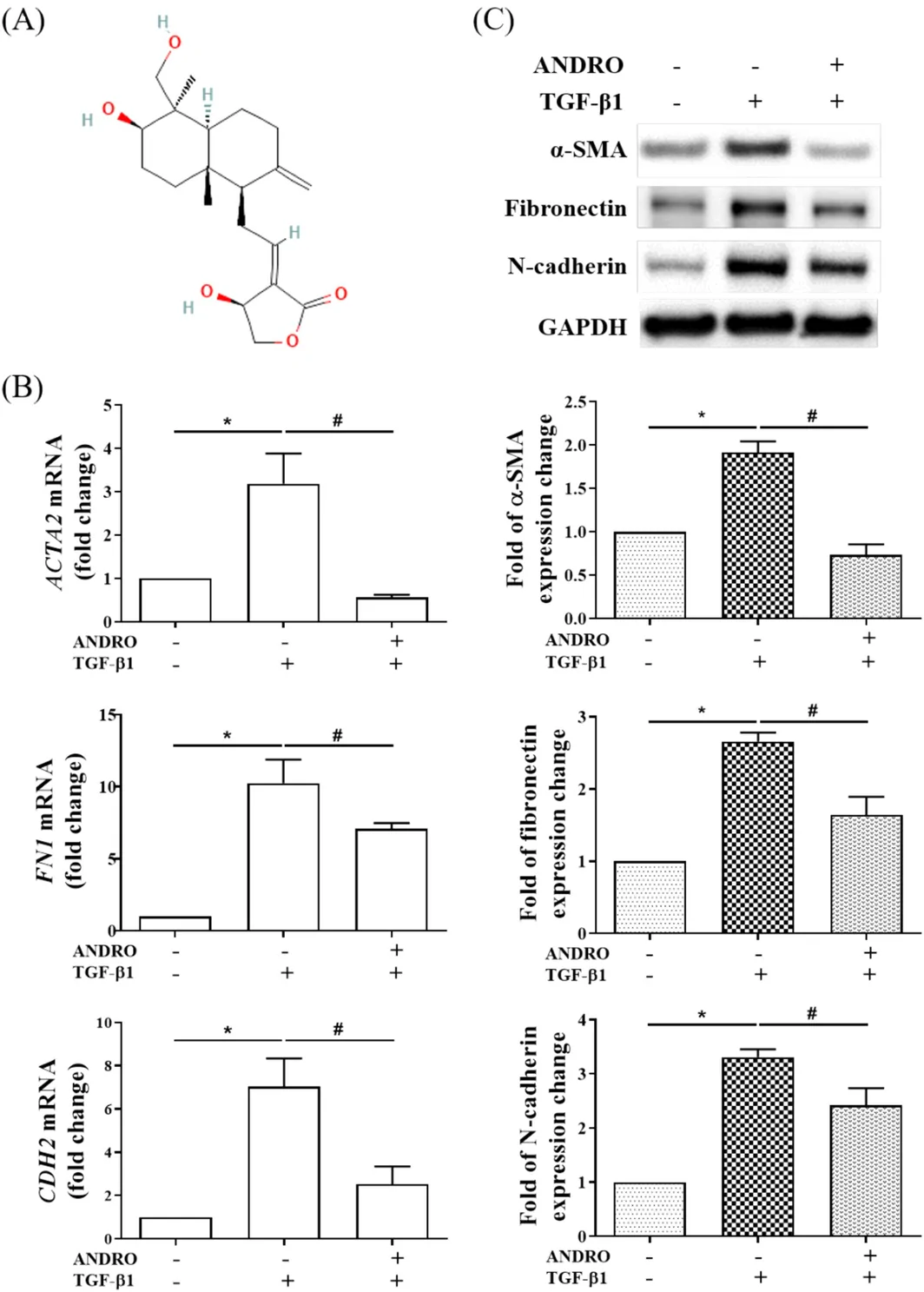
Effects of ANDRO on the expression of TGF‐β1‐induced gene and protein associated with fibrogenesis. MRC5 cells were pretreated with or without ANDRO (10 μM) for 4 h. Subsequently, exogenous TGF‐β1 (4 ng/mL) was added to the medium for 48 h. (A) The chemical structure of ANDRO was generated based on data from PubChem (CID: 5318517). (B) Gene expression of *ACTA2*, *FN1*, and *CDH2* was detected through RT‐qPCR, and *GAPDH* was used as a loading control. (C) Protein expression of α‐SMA (encoded by *ACTA2*), fibronectin (encoded by *FN1*), and N‐cadherin (encoded by CDH2) was measured by Western blotting. One‐way ANOVA was used to analyze differences among groups, and the Tukey's multiple comparison test was performed for post hoc analysis. Data are presented as the mean ± SEM of three independent experiments. *p < 0.05 versus control. #*p* < 0.05 versus TGF‐β1 treatment.

Under the same treatment conditions described above, comprehensive gene expression profiles were obtained by RNA‐seq to investigate the mechanisms by which ANDRO attenuated TGF‐β1‐induced fibrogenesis. DEGs involved in these molecular targets and pathways were determined through bioinformatics, with the results revealing that exogenous TGF‐β1 stimulation significantly modulated 3811 genes. Furthermore, pretreatment with ANDRO significantly attenuated the upregulation or downregulation of 596 of these genes modulated by TGF‐β1. These 596 genes were used as candidate genes, with ShinyGO 0.76 being used for functional enrichment analysis, STRING software used to visualize protein–protein interactions, RT‐qPCR used to validate gene expression changes, and Western blotting used to detect protein expression. Additionally, changes in cell function were confirmed through subsequent molecular biological experiments. Figure 2 presents the experimental design and the analysis workflow for these RNA‐seq studies.

**FIGURE 2:**
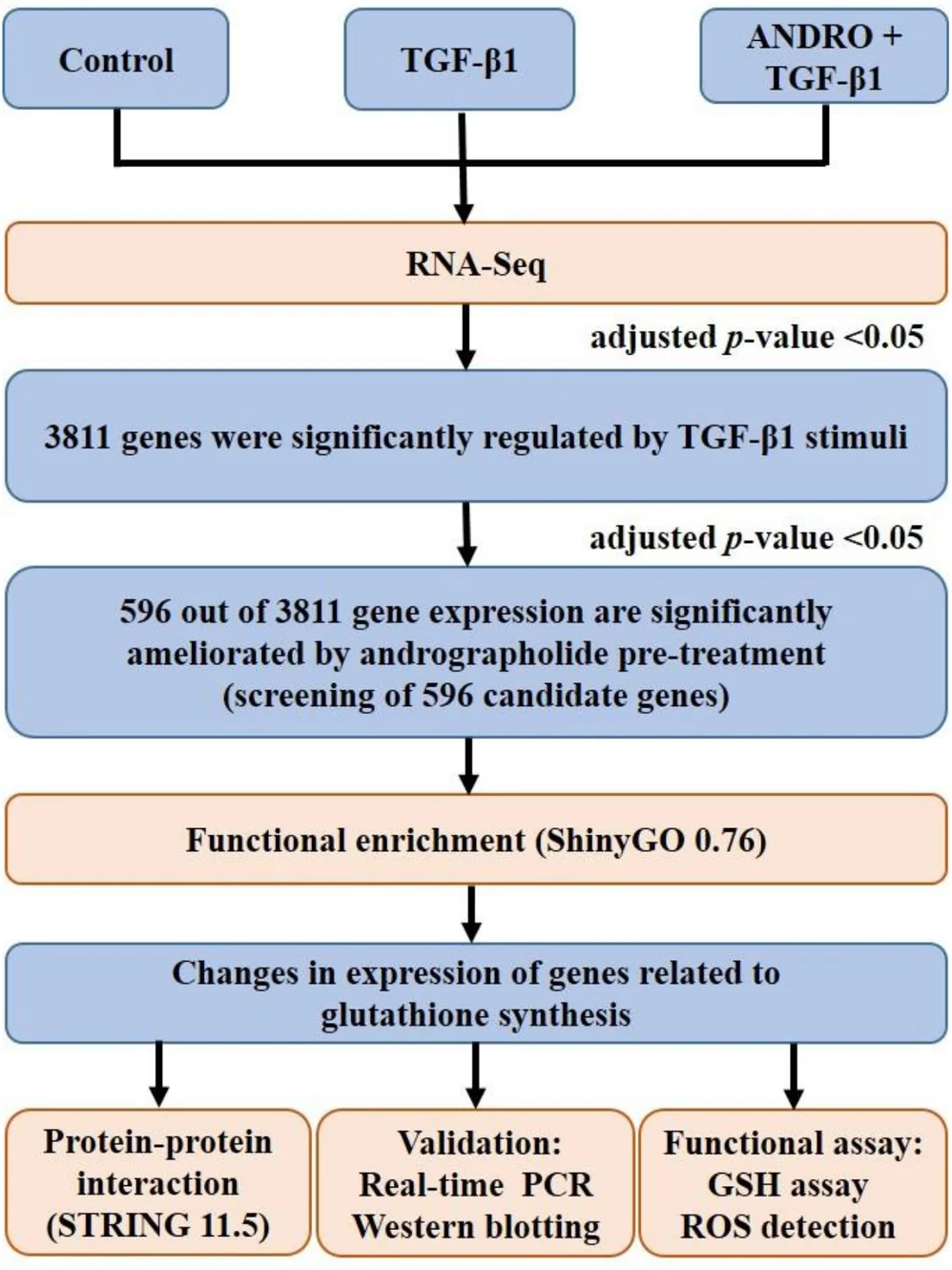
Schematic diagram of study design and analysis strategy. MRC5 cells were treated with or without 10 μM ANDRO for 4 h. Subsequently, 4 ng/mL of TGF‐β1 was added to the culture medium for 48 h. Total RNA was extracted and sequenced using RNA‐Seq to obtain gene expression profiles. We screened 3811 DEGs between the control and TGF‐β1 groups. Of these 3811 DEGs, 596 were significantly reversed by ANDRO treatment and were selected for further functional enrichment analysis using the ShinyGO 0.76. Significantly altered genes were analyzed, and the corresponding protein–protein interaction networks were generated using STRING 11.5. Validation of gene expression was performed by RT‐qPCR. Protein expression was detected by Western blotting. GSH level and ROS production were detected using the GSH assay and DCFH‐DA staining, respectively.

### 3.2 ANDRO Reversed TGF‐β1‐Perturbed Gene Profile Associated With GSH Metabolism

The antifibrotic pathways through which ANDRO attenuated TGF‐β1‐induced fibrogenesis were identified via Kyoto Encyclopedia of Genes and Genomes (KEGG) pathway enrichment analysis of the 596 candidate genes (ShinyGO 0.76). The results indicated that GSH metabolism was among the top 10 significantly enriched KEGG pathways containing the largest number of differentially expressed candidate genes. Seven candidate genes, namely *GCLM*, *GSTA4*, *MGST1*, *GSR*, *MGST2*, *GSTM3*, and *G6PD*, were involved in GSH metabolism (Table 2). Heatmap analysis of these seven genes was performed, with the results shown in Figure 3A. In addition, a protein–protein interaction network of these genes was constructed using STRING software to visually illustrate interactions among the encoded proteins (Figure 3B).

**TABLE 2:** Seven DEGs were involved in the GSH metabolism.

Gene ID	Symbol	Description	TPM			*p*_adj_		Regulation	
			Control	TGF‐β1	ANDRO vs. TGF‐β1	TGF‐β1 vs. Control	ANDRO vs. TGF‐β1	TGF‐β1 vs. Control	ANDRO vs. TGF‐β1
ENSG00000023909	GCLM	Glutamate‐cysteine ligase modifier subunit	50.34776	25.74703	78.2111	0.000546	5.85E‐11	Down	Up
ENSG00000170899	GSTA4	Glutathione S‐transferase alpha 4	22.45566	10.89231	28.1984	0.004094	1.43E‐05	Down	Up
ENSG00000008394	MGST1	Microsomal glutathione S‐transferase 1	89.59703	33.61585	125.1987	1.03E‐08	6.79E‐16	Down	Up
ENSG00000104687	GSR	Glutathione‐disulfide reductase	52.90716	31.69284	66.3274	0.02044	4.76E‐05	Down	Up
ENSG00000085871	MGST2	Microsomal glutathione S‐transferase 2	7.46932	3.271742	6.902533	0.007929	0.015894	Down	Up
ENSG00000134202	GSTM3	Glutathione S‐transferase mu 3	70.56841	31.42801	72.03378	9.71E‐06	2.05E‐06	Down	Up
ENSG00000160211	G6PD	Glucose‐6‐phosphate dehydrogenase	273.1274	125.185	308.2751	1.04E‐05	5.68E‐08	Down	Up

*Note:* GSTA4, GSTM3, MGST1, and MGST2 are members of the GST family.

Abbreviation: TPM: transcript per million.

**FIGURE 3:**
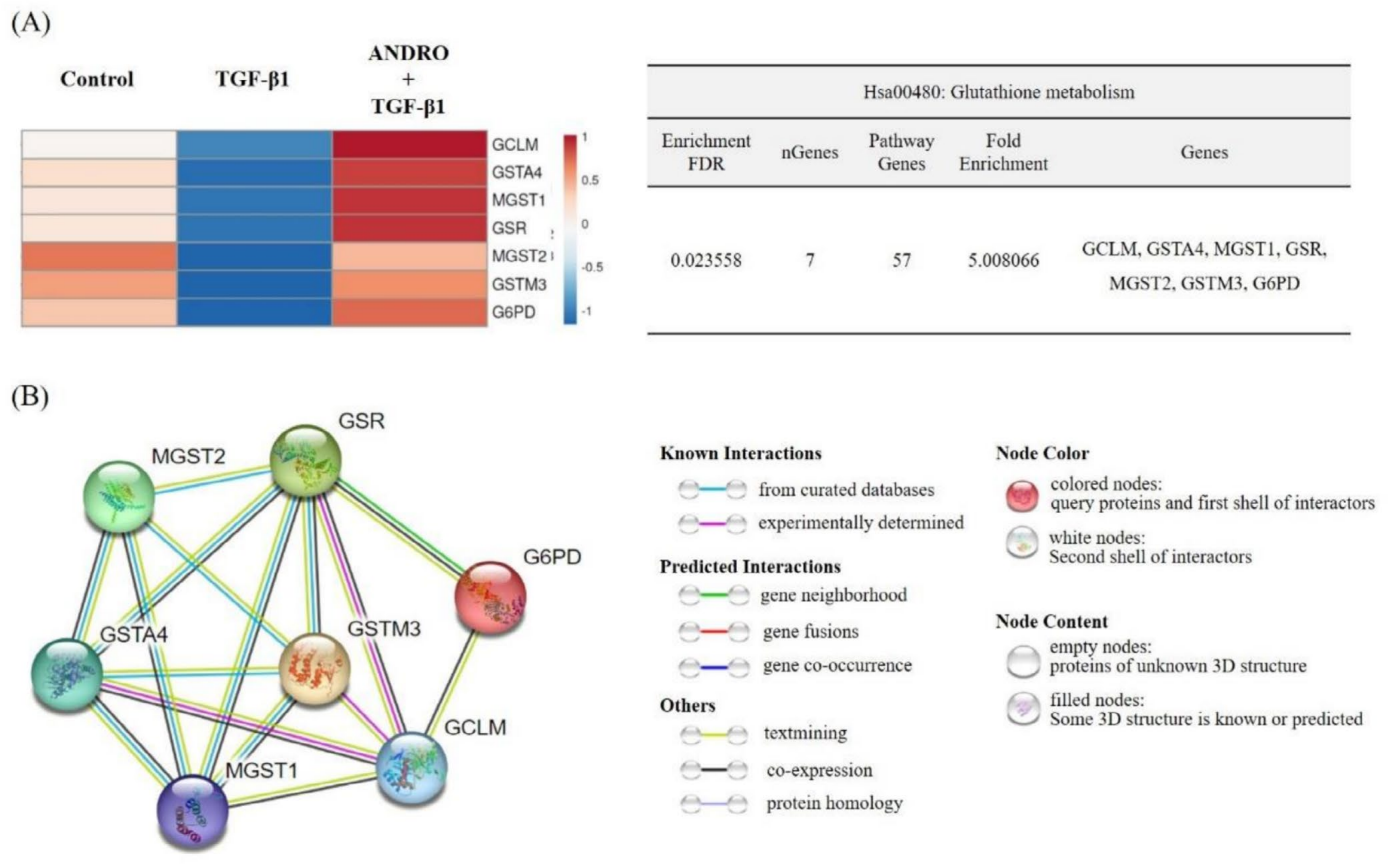
Clustering analysis of candidate DEGs associated with GSH metabolism in MRC5 cells. (A) Heatmap showing the hierarchical clustering of genes (*GCLM*, *GSTA4*, *MGST1*, *GSR*, *MGST2*, *GSTM3*, and *G6PD*) associated with GSH metabolism. (B) Network cluster analysis of protein–protein interactions for genes involved in GSH metabolism was performed using STRING software.

### 3.3 ANDRO Restored TGF‐β1‐Perturbed Expression of Genes and Proteins Related to GSH Metabolism

To explore the involvement of GSH metabolism in ANDRO's antifibrotic effects, we selected three genes representing key regulatory nodes of GSH metabolism—GCLM, *G6PD*, and *GSR—*from seven candidate genes for validation. *GCLM* regulates the rate‐limiting step in GSH biosynthesis [25]; *G6PD* generates NADPH to support GSH regeneration [26]; and *GSR* maintains the intracellular GSH/GSSG homeostasis [27].

MRC5 cells were pretreated with or without ANDRO (10 μM) for 4 h, followed by stimulation with exogenous TGF‐β1 (4 ng/mL) for 48 h. RT‐qPCR analysis revealed that ANDRO restored the TGF‐β1‐inhibited expression of *GCLM*, *G6PD*, *and GSR* (Figure 4A), consistent with the RNA‐seq results. To confirm that this effect was not specific to MRC5 cells, the same treatment was applied to WI‐38 cells, yielding similar gene expression patterns (Figure 4B). Consistent with these transcriptional findings, Western blot analysis demonstrated that ANDRO also reversed the TGF‐β1‐suppressed protein expression of GCLM, G6PD, and GSR in MRC5 (Figure 5A) and WI‐38 (Figure 5B). In addition, in the absence of TGF‐β1 stimulation, RT‐qPCR analysis showed that ANDRO significantly induced the basal expression of *GCLM*, *G6PD*, and *GSR* in MRC5 cells. In parallel experiments using WI‐38 cells, ANDRO significantly increased the expression of *GCLM* and *GSR*, while *G6PD* showed a modest upward trend that did not reach statistical significance (Figure S2).

**FIGURE 4:**
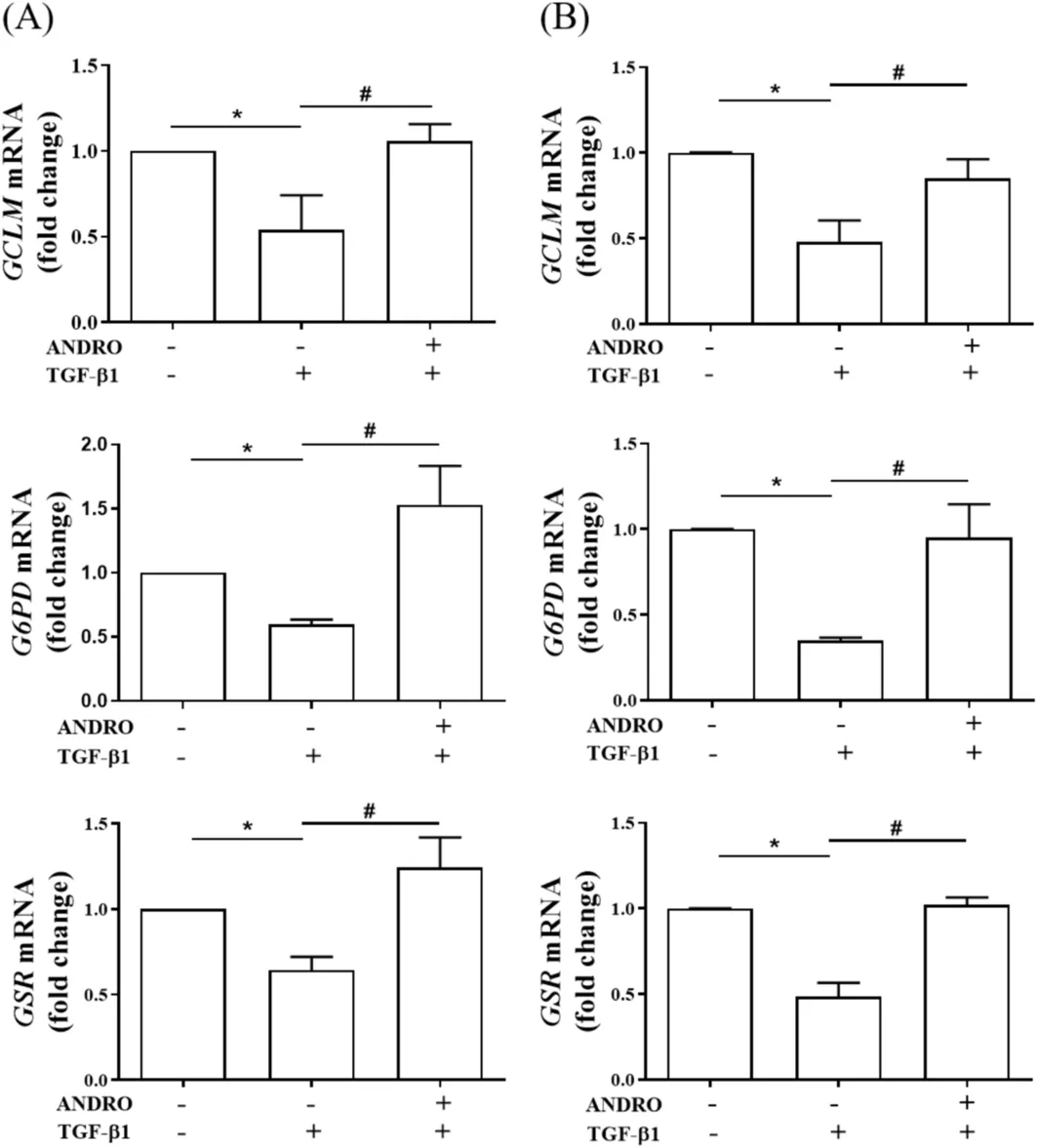
Validation of gene expression. Cells were pretreated with or without ANDRO (10 μM) for 4 h. Subsequently, exogenous TGF‐β1 (4 ng/mL) was added to the medium for 48 h. Gene expression of *GCLM*, *GSR*, and *G6PD* was measured in (A) MRC5 cells and (B) WI‐38 cells through RT‐qPCR. One‐way ANOVA was used to analyze differences among groups, and the Tukey's multiple comparison test was performed for post hoc analysis. Data are presented as the mean ± SEM of three independent experiments. *p < 0.05 versus control. #*p* < 0.05 versus TGF‐β1 treatment.

**FIGURE 5:**
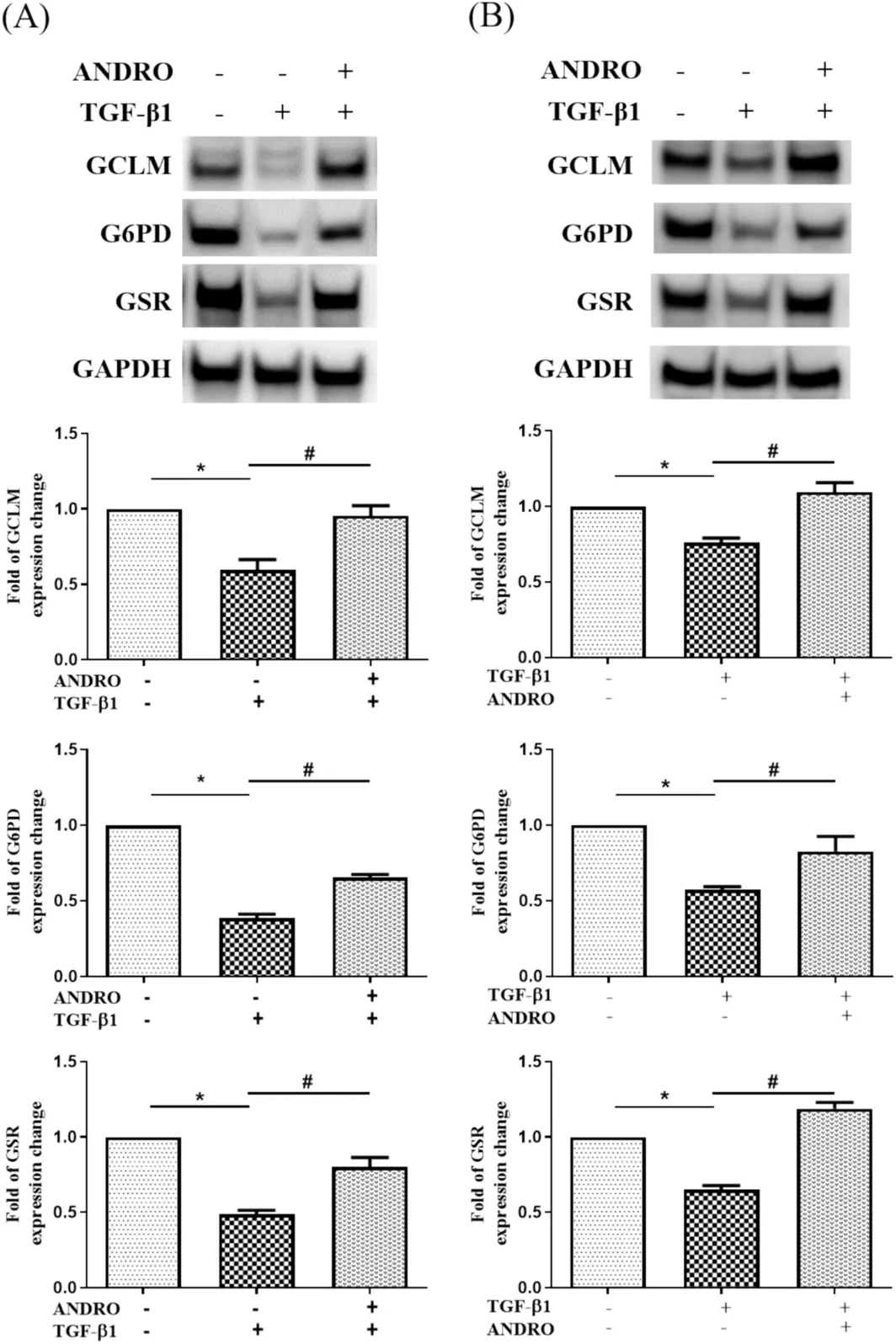
Effects of ANDRO on expression of proteins related to GSH metabolism. MRC5 and WI‐38 cells were pretreated with or without ANDRO (10 μM) for 4 h, followed by the addition of exogenous TGF‐β1 (4 ng/mL) to the medium for 48 h. Expressions of GSH metabolism‐related proteins, including GCLM, GSR, and G6PD, were examined in (A) MRC5 cells and (B) WI‐38 cells by Western blotting. One‐way ANOVA was used to analyze differences among groups, and the Tukey's multiple comparison test was performed for post hoc analysis. Data are presented as the mean ± SEM of three independent experiments. *p < 0.05 versus control. #*p* < 0.05 versus TGF‐β1 treatment.

### 3.4 ANDRO Increased TGF‐β1‐Inhibited Total GSH Level

The potential of ANDRO to modulate GSH levels was investigated after confirming the gene and protein expression associated with GSH metabolism. MRC5 and WI‐38 cells were pretreated with or without ANDRO (10 μM) for 4 h. Subsequently, exogenous TGF‐β1 (4 ng/mL) was added to the medium for 48 h, then GSH levels were measured using the GSH assay. The results revealed that ANDRO reversed the TGF‐β1‐inhibited total GSH level in both MRC5 cells and WI‐38 cells (Figure 6).

**FIGURE 6:**
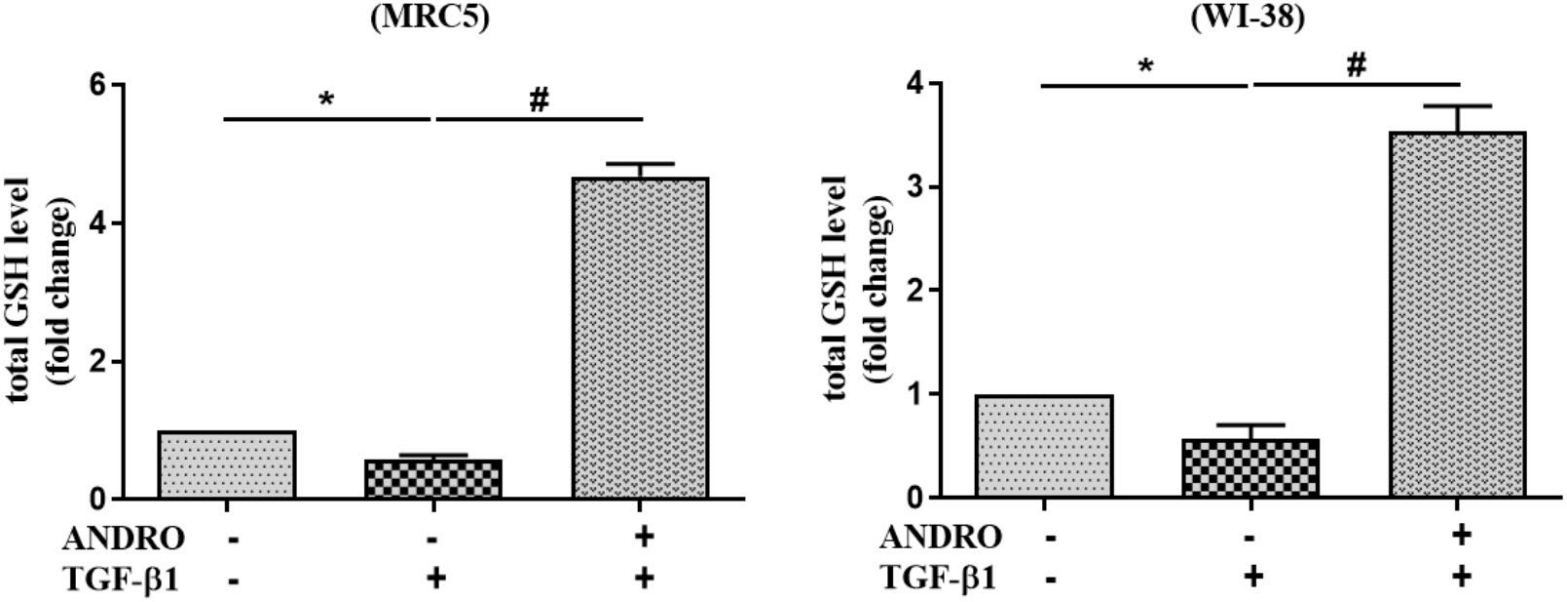
Effects of ANDRO on TGF‐β1‐inhibited GSH level. MRC5 and WI‐38 cells were pretreated with or without ANDRO (10 μM) for 4 h. Subsequently, exogenous TGF‐β1 (4 ng/mL) was added to the medium for 48 h. Total GSH level was measured in MRC5 and WI‐38 cells by GSH assay. One‐way ANOVA was used to analyze differences among groups, and the Tukey's multiple comparison test was performed for post hoc analysis. Data are presented as the mean ± SEM of three independent experiments. *p < 0.05 versus control. #*p* < 0.05 versus TGF‐β1 treatment.

### 3.5 ANDRO Suppressed TGF‐β1‐Induced ROS Production

Given that GSH serves as a major intracellular antioxidant protecting cells from oxidative stress caused by ROS, we next examined the effects of ANDRO on ROS production using DCFH‐DA staining. MRC5 and WI‐38 cells were pretreated with or without ANDRO (10 μM) for 4 h, followed by exposure to exogenous TGF‐β1 (4 ng/mL) for 48 h. ROS levels were assessed through the DCFH‐DA assay, and the results revealed that ANDRO markedly inhibited TGF‐β1‐induced ROS accumulation in both MRC5 and WI‐38 cells (Figure 7).

**FIGURE 7:**
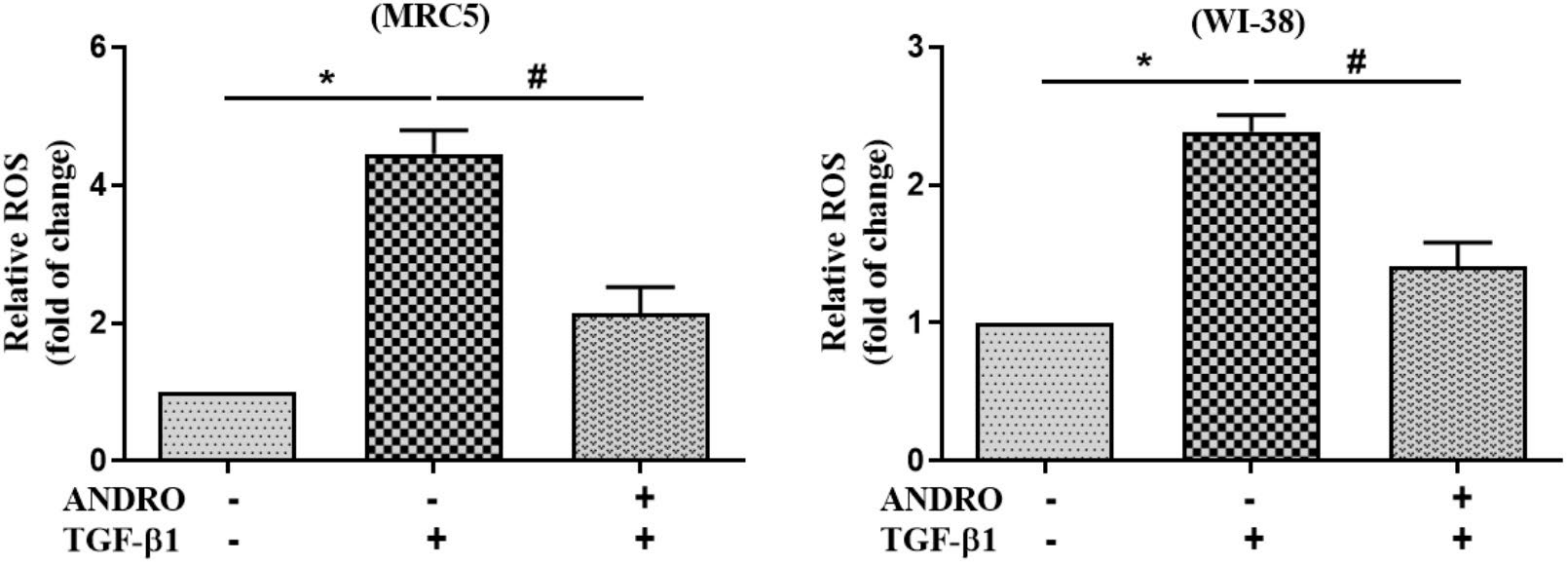
Effects of ANDRO on TGF‐β1‐induced ROS production. MRC5 and WI‐38 Cells were pretreated with or without ANDRO (10 μM) for 4 h. Subsequently, exogenous TGF‐β1 (4 ng/mL) was added to the medium for 48 h. ROS levels were detected in MRC5 and WI‐38 cells by DCFH‐DA staining, and fluorescence intensity was measured using a microplate reader. One‐way ANOVA was used to analyze differences among groups, and the Tukey's multiple comparison test was performed for post hoc analysis. Data are presented as the mean ± SEM of three independent experiments. *p < 0.05 versus control. #*p* < 0.05 versus TGF‐β1 treatment.

Based on these findings, we constructed a schematic model to illustrate the potential mechanism by which ANDRO mitigates TGF‐β1‐induced fibrogenesis in human lung fibroblasts. The model summarizes the central role of GSH metabolism and oxidative stress regulation in mediating the antifibrotic effects of ANDRO (Figure 8).

**FIGURE 8:**
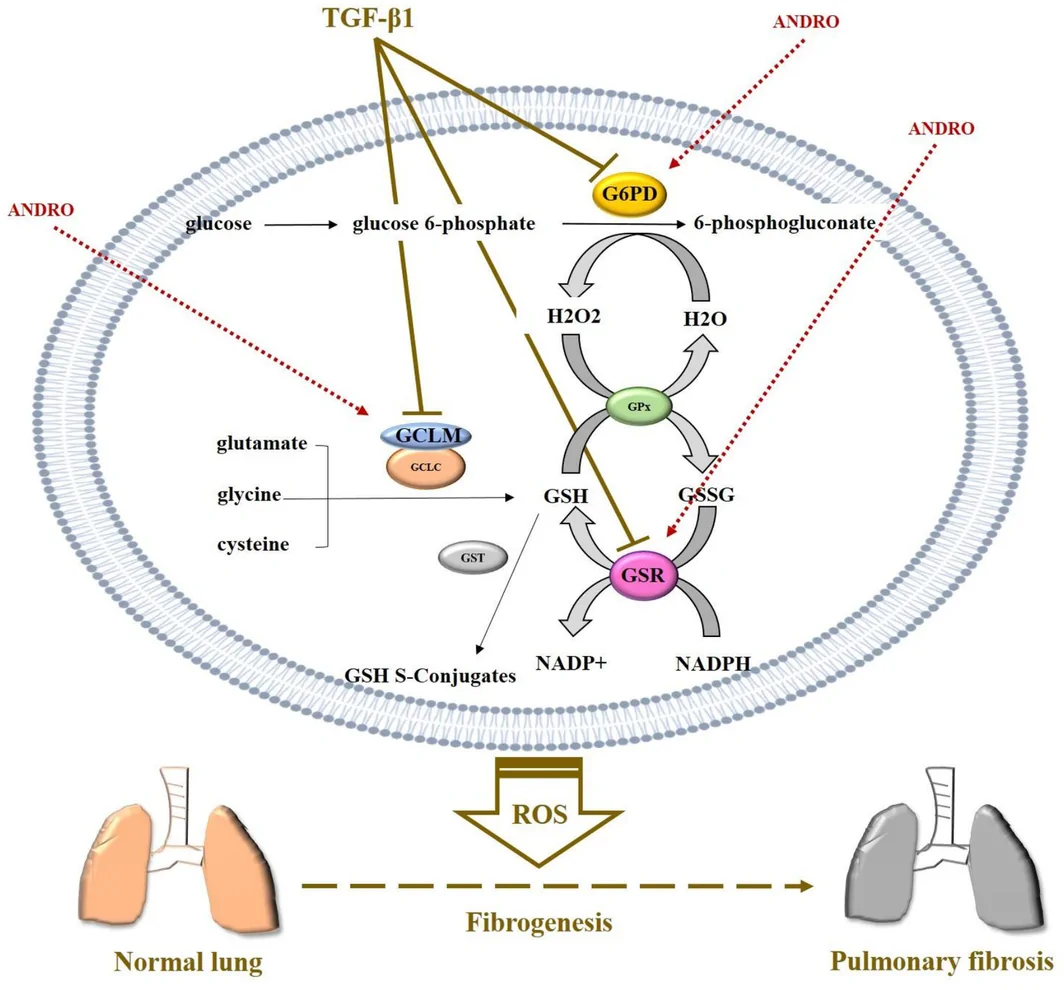
Schematic diagram of ANDRO attenuating TGF‐β1‐induced fibrogenesis by modulating GSH metabolism in human lung fibroblasts. TGF‐β1 disrupts GSH homeostasis by downregulating key regulators of GSH metabolism, including GCLM, GSR, and G6PD, leading to excessive ROS accumulation and fibrotic responses. ANDRO reversed TGF‐β1‐modulated expression of genes and proteins associated with GSH metabolism. In addition, ANDRO restored TGF‐β1‐inhibited total GSH level and inhibited TGF‐β1‐induced ROS production. The involvement of ROS in TGF‐β1‐induced fibrogenic responses is supported by previous studies [28].

## 4 Discussion

TGF‐β1 is a key profibrotic cytokine, and ANDRO has been reported to alleviate pulmonary fibrosis [24, 29]. In this study, we investigated how ANDRO attenuates TGF‐β1‐induced fibrogenesis in human lung fibroblasts using RNA‐seq. Our results revealed that GSH metabolism was among the top 10 significantly enriched KEGG pathways, comprising key genes such as *GCLM*, *GSTA4*, *MGST1*, *GSR*, *MGST2*, *GSTM3*, and *G6PD*. Validation of these genes through RT‐qPCR, along with Western blot analysis of the corresponding proteins, revealed that ANDRO reversed the TGF‐β1‐modulated gene and protein expression associated with GSH metabolism in human lung fibroblasts. In addition, ANDRO restored TGF‐β1‐inhibited total GSH levels and inhibited TGF‐β1‐induced ROS production. These results indicate that ANDRO may alleviate pulmonary fibrosis by modulating GSH metabolism and scavenging excess ROS. These findings are in line with previous reports showing that ANDRO activates antioxidant signaling pathways, including Nrf2/HO‐1 via p38 MAPK and ERK, as well as PI3K/AKT/mTOR [19, 20, 21], which may underlie its ability to restore GSH metabolism and reduce ROS in human lung fibroblasts.

Notably, our results demonstrate that in the absence of TGF‐β1 stimulation, ANDRO treatment alone significantly suppressed the basal expression of fibrosis‐associated markers (*ACTA2*, *FN1*, and *CDH2*), while upregulating GSH metabolism‐related genes (*GCLM*, *GSR*, and *G6PD*) in MRC5 cells. In parallel experiments with WI‐38 cells, we obtained similar conclusions to those with MRC5 cells, except that the inhibitory effect of ANDRO on *CDH2* and the promoting effect on *G6PD* expression did not reach statistical significance. These findings suggest that ANDRO exerts intrinsic regulatory effects on fibroblast activation status and redox‐related gene expression, beyond merely antagonizing TGF‐β signaling. Such basal modulation may contribute to maintaining fibroblasts in a less activated state and potentially enhancing their resistance to profibrotic and oxidative stress stimuli.

Oxidative stress, defined as an imbalance between elevated ROS and insufficient antioxidant defenses, plays a central role in the pathogenesis of pulmonary fibrosis. A significant increase in the ability of inflammatory cells in the bronchoalveolar lavage fluid of patients with pulmonary fibrosis to release hydrogen peroxide and myeloperoxidase has been detected [30, 31]. Oxidative stress may directly or indirectly lead to alveolar epithelial cell injury and fibroblast proliferation that consequently leads to excessive deposition of ECM proteins, and the maintenance of a high intracellular level of reduced GSH is considered to be important for providing the reducing environment to protect against oxidative stress [30, 32]. Importantly, our study provides direct mechanistic evidence in human lung fibroblasts that ANDRO attenuates oxidative stress and modulates GSH metabolism, complementing previous observations in rodent models and other cell types, and reinforcing its antifibrotic potential.

Previous studies have demonstrated the modulatory effect of ANDRO on GSH homeostasis in various models. For example, in non–small‐cell lung cancer cells, ANDRO inhibited proliferation and metastasis by inducing ferroptosis. The mechanism involved alterations in GSH biosynthesis, lipid peroxidation, and iron metabolism [33]. In addition, ANDRO induced cytotoxicity in hepatoma cells by targeting the crosstalk between JNK activation and cellular GSH homeostasis [34]. Furthermore, ANDRO inhibited the activation of NADPH oxidase and upregulated *GCLM* expression in EA.hy926 endothelial‐like cells through the PI3K/Akt pathway [21]. In addition, ANDRO significantly increased the GSH to GSSG ratio in a mouse model of bleomycin‐induced pulmonary fibrosis [35]. To our knowledge, this is the first study to demonstrate that ANDRO attenuated TGF‐β1‐induced fibrogenesis by modulating GSH metabolism in human lung fibroblasts.

TGF‐β1 increases ROS production in various cell types, and in turn, ROS activates TGF‐β1, contributing to many TGF‐β1‐mediated fibrotic effects [28]. GSH is a crucial antioxidant, and its concentration in the lungs is decreased in both fibrotic diseases and experimental fibrosis models. A previous study demonstrated that by elevating TGF‐β1 expression in the lungs of mice to levels comparable to those found in lung fibrotic diseases through the intranasal administration of a recombinant adenovirus of active TGF‐β1 (AdTGF‐β1^223/225^), the expression of glutamate cysteine ligase catalytic (GCLC) and modifier (GCLM) subunits was suppressed, both of which are essential for *de novo* GSH synthesis, resulting in a reduction in GSH levels [14, 36]. Furthermore, TGF‐β1 induced severe pulmonary fibrosis when GSH was depleted in lung epithelial cells.

ANDRO is a natural product extracted from *A. paniculata* and exhibits various pharmacological activities. The molecular mechanisms underlying the diverse pharmacological activities of ANDRO have been partially elucidated, and ANDRO has even entered clinical trials for primary progressive multiple sclerosis, colorectal cancer, acute tonsillitis, acute bronchitis, esophageal squamous cell carcinoma, and migraine [18]. Although clinical trials for pulmonary fibrosis are still lacking, existing evidence supports the clinical potential of ANDRO. Further elucidation of its molecular mechanisms will aid in accelerating the clinical use of ANDRO products for the prevention and treatment of pulmonary fibrosis.

In conclusion, this in vitro study demonstrates that ANDRO attenuates TGF‐β1‐induced fibrogenic responses in human lung fibroblasts by restoring GSH metabolism and reducing ROS accumulation. Although in vivo validation is required to confirm its therapeutic efficacy and safety, we acknowledge that this study was limited to in vitro experiments using only two fibroblast cell lines and the lack of in vivo validation. Nevertheless, these findings provide novel mechanistic insight and support the potential of ANDRO as a preventive and therapeutic agent for pulmonary fibrosis.

## Funding

This study was supported by the National Science and Technology Council, Taiwan (grant numbers NSTC 112‐2314‐B‐037‐063‐ and NSTC 114‐2320‐B‐037‐013‐MY3), Kaohsiung Medical University Hospital (grant numbers KMUH112‐2R47, KMUH113‐3M28 and KMUH114‐4R45), and the Chi Mei Medical Center, Liouying (grant numbers CLFHR11234 and CLFHR11303).

## Conflicts of Interest

The authors declare no conflicts of interest.

## Supporting information


**Figure S1:** Effects of ANDRO on the expression of *ACTA2*, *FN1*, and *CDH2* was detected through RT‐qPCR, and *GAPDH* was used as a loading control in (A) MRC5 and (B) WI‐38 cells. Statistical comparisons between control and ANDRO groups were performed using an unpaired Student's t test with Welch's correction. Data are presented as the mean ± SEM of three independent experiments. A *p* value < 0.05 was considered statistically significant.
**Figure S2:** Effects of ANDRO on the expression of *GCLM*, *G6PD*, and *GSR* was detected through RT‐qPCR, and *GAPDH* was used as a loading control in (A) MRC5 and (B) WI‐38 cells. Statistical comparisons between control and ANDRO groups were performed using an unpaired Student's *t* test with Welch's correction. Data are presented as the mean ± SEM of three independent experiments. A *p* value < 0.05 was considered statistically significant.

## Data Availability

The data that support the findings of this study are available from the corresponding author upon reasonable request.
