# Supplementary material for: Andrographolide Modulates Fibrogenic and Oxidative Stress Responses in Human Lung Fibroblasts
Source: Kaohsiung J Med Sci. 2026 Apr 22;42(11):e70218. doi: 10.1002/kjm2.70218 (PMC13399617; doi:10.1002/kjm2.70218)
Supplement: Supplementary file 1 [file KJM2-42-e70218-s001.docx]

**Supplementary data**

**Supplementary Figure 1.** Effects of ANDRO on the expression of *ACTA2*, *FN1*, and *CDH2* was detected through RT-qPCR, and *GAPDH* was used as a loading control in (A) MRC5 and (B) WI-38 cells. Statistical comparisons between control and ANDRO groups were performed using an unpaired Student’s t test with Welch’s correction. Data are presented as the mean ± SEM of three independent experiments. A *p* value < 0.05 was considered statistically significant.

**Supplementary Figure 2.** Effects of ANDRO on the expression of *GCLM*, *G6PD*, and *GSR* was detected through RT-qPCR, and *GAPDH* was used as a loading control in (A) MRC5 and (B) WI-38 cells. Statistical comparisons between control and ANDRO groups were performed using an unpaired Student’s *t* test with Welch’s correction. Data are presented as the mean ± SEM of three independent experiments. A *p* value < 0.05 was considered statistically significant.
